# Mycotoxin Production and the Relationship between Microbial Diversity and Mycotoxins in *Pyrus bretschneideri* Rehd cv. Huangguan Pear

**DOI:** 10.3390/toxins14100699

**Published:** 2022-10-11

**Authors:** Huimin Li, Yang Zhang, Congcong Gao, Qi Gao, Yudou Cheng, Min Zhao, Junfeng Guan

**Affiliations:** 1 School of Landscape and Ecological Engineering, Hebei Engineering University, Handan 056021, China; 2 Institute of Biotechnology and Food Science, Hebei Academy of Agricultural and Forestry Sciences, Shijiazhuang 050051, China; 3 Key Laboratory of Plant Genetic Engineering Center of Hebei Province, Shijiazhuang 050051, China

**Keywords:** microbiome, mycotoxin, ‘Huangguan’ pear, postharvest rotten

## Abstract

Mycotoxins are generated by a series of fungal pathogens in postharvest fruit, resulting in serious health threat to consumers and great economic loss to the fruit storage industry. The microbial differences between rotten and healthy fruit during storage and their relationship with mycotoxin production have not been fully studied. In this study, differences in microbial diversity between rotten and healthy fruit after 30 days of storage at ambient temperature were investigated using high-throughput sequencing technology in ‘Huangguan’ pear (*Pyrus bretschneideri* Rehd cv. Huangguan) harvested from five different producing regions of Hebei province, China. The bacterial genus *Gluconobacter* was much more abundant in rotten fruit (76.24%) than that in healthy fruit (32.36%). In addition, *Komagataeibacter* and *Acetobacter* were also relatively higher in abundance in rotten fruit. In contrast, bacterial genera *Pantoea*, *Alistipes*, *Muribaculaceae*, *Lactobacillus*, and *Ruminococcaceae*_UCG were found to be more abundant in healthy fruit. Fungal genera including *Botryosphaeria*, *Colletotrichum*, *Valsa*, *Alternaria*, *Rosellinia*, *Fusarium*, and *Trichothecium* were found to be abundant in rotten fruit. The results of principal coordinate analysis (PCoA) showed that there were significant differences in the microbial diversity of different regions. PAT (patulin) was detected in all rotten fruit samples, while tenuazonic acid (TeA), alternariol (AOH), and alternariolmonomethyl ether (AME) were only detected in samples collected from one region (Weixian). Canonical correlation analysis (CCA) and Pearson correlation analysis showed that the abundance of *Alistipes* and *Pantoea* were negatively correlated with the contents of PAT, suggesting that bacterial genera *Alistipes* and *Pantoea* have potential in reducing mycotoxin production in ‘Huangguan’ pear.

## 1. Introduction

Postharvest storage of fruits faces many challenges, among which fruit rot caused by plant pathogens is one of the major threats, which often causes great economic losses. The major agents leading to the spoilage of fruits are those attributed to fungal pathogens, such as *Alternaria*, *Penicillium*, *Aspergillus*, *Botrytis*, *Rhizopus*, *Colletotrichum*, and *Monilinia*, which have been widely reported [1,2,3,4,5,6].

Fruit rot caused by fungi is often accompanied by mycotoxin contamination. Mycotoxins are present in fruits even after the fungal hyphae have been eliminated, and they can spread to healthy tissues. Patulin (PAT) is considered to be the most important mycotoxin in fruits worldwide [7], and it is found to be difficult to degrade and can be detected not only in fruits but also in processed fruit products [8]. Alternaria toxins are secondary metabolites mainly produced by *Alternaria*, of which tenuazonic acid (TeA), alternariol (AOH), and alternariolmonomethyl ether (AME) are deeply studied and widely reported [9]. Temperature, a_w_, relative humidity, pH, fungal strain, and substrate are generally considered to be the main factors that affect mycotoxin production [10]. Biological degradation also affects the content of mycotoxins in a certain environment. [11]. Interestingly, non-toxic fungal metabolites can sometimes play a synergistic function with toxic metabolites, so the co-existence of multiple mycotoxins may enhance toxicity, and the characteristics of microbial communities need to be focused on as well [12]. Mycotoxins pose a serious threat to human health, and may cause cancer and malformation in some cases [13]. Acute toxicity caused by mycotoxins results in death, while chronic toxicity results in cancers, immune suppression, and other generally irreversible effects [13,14]. Therefore, it is of great significance to take appropriate measures to reduce toxin production in fruits.

Pear (*Pyrus bretschneideri* R.) is one of the most important fruits produced in China, which has been threatened by pathogens during postharvest storage [15]. Many studies have shown that postharvest pear fruit diseases are caused by a variety of pathogens [16,17,18]. However, the relationship between pathogenic fungi and bacteria and their correlation with toxins have not been revealed. Microbiome technology is a new and effective way to show the composition, structure, and diversity of microbial communities in various environments [19], and its application has broken the limitation of traditional methods such as microbial isolation, greatly improved the utilization of microbial resources, and become the most important frontier and hotspot in microbial research [20]. Postharvest microbiome research is considered to be a promising approach to reveal the issues of postharvest fruit quality, safety, and sustainability [21]. To date, more and more studies on fruit microbiome have been reported, but research on postharvest microbiome is still insufficient. The study of postharvest microbiome offers important opportunities to develop a theoretical basis for the prevention and control of postharvest fruit diseases [22].

In the present study, ‘Huangguan’ pear (*Pyrus bretschneideri* Rehd cv. Huangguan) fruit was collected from five main production areas of Hebei province, China, and subsequently stored at ambient temperature for 30 days. ‘Huangguan’ pear is a well-known medium-maturity cultivar in north China, containing important nutrients including proteins, carotene, vitamin B1 and B2, and malic acid, and has a wide consumption market in China [23,24]. The differences in microbial communities and mycotoxins between healthy and rotten fruit were compared and analyzed. This study aims to investigate the relationship between mycotoxin production and microbial composition, and to further explore the microbial factors affecting the mycotoxin production in this pear fruit.

## 2. Results

### 2.1. Microbial Community Composition in Pear Fruits

**Figure 1:**
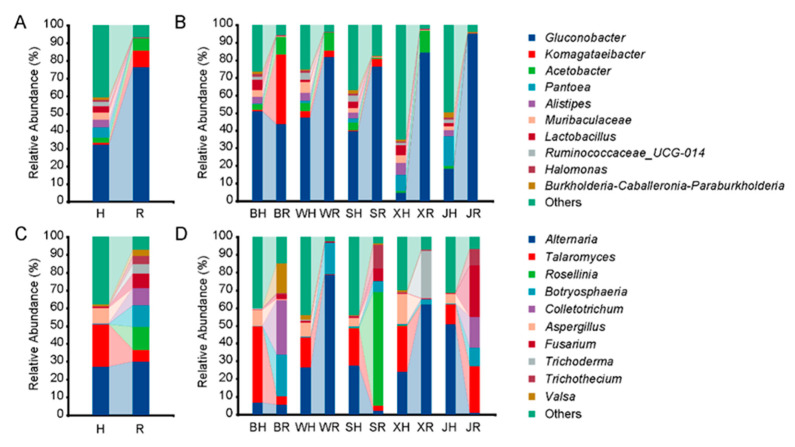
Microbial composition in healthy (H) and rotten (R) fruit of ‘Huangguan’ pear. (**A**) Overall bacterial composition; (**B**) bacterial composition in five producing regions; (**C**) overall fungal composition; (**D**) fungal composition in five producing regions. Healthy fruits from Botou, Weixian, Shenzhou, Xinji, and Jinzhou were named BH, WH, SH, XH, and JH, while rotten fruits were named BR, WR, SR, XR, and JR, respectively.

A total of 5,808,827 high-quality sequences were obtained after being denoised, merged, and de-duplicated in bacteria, which were assigned to 12,790 amplicon sequence variants (ASVs). The minimum sequence amount was 82,885, while the maximum was 143,484 across all the samples. The composition of bacterial communities differed significantly between healthy and rotten pear fruit (Figure 1A). The relative abundance of *Gluconobacter* accounted for 32.36% in healthy fruit, whereas 76.24% in rotten fruit. In addition, *Pantoea*, *Alistipes*, *Muribaculaceae*, *Lactobacillus*, and *Ruminococcaceae*_UCG were relatively abundant in healthy fruit, but rarely present in rotten fruit. In contrast, *Komagataeibacter* and *Acetobacter* were more abundant in rotten fruit. We further compared the changes in bacterial composition in five main producing regions of ‘Huangguan’ pear, and it was found that the relative abundance of *Gluconobacter* was significantly enriched in rotten fruit from most producing regions, except one region, Botou, where the main bacteria increased was *Komagataeibacter* (Figure 1B).

For fungi, a total of 4,135,114 high-quality sequences (minimum, 51,499; maximum, 152,430) were assigned to 1518 fungal ASVs. The most dominant fungi were *Alternaria* (27.1%), *Talaromyces* (23.6%), and *Aspergillus* (8.7%) in healthy fruit (Figure 1C). In contrast, *Alternaria* (30.02%), *Talaromyces* (6.73%), *Rosellinia* (12.81%), *Botryosphaeria* (12.06%), *Colletotrichum* (9.58%), *Fusarium* (7.88%), *Trichoderma* (5.43%), *Trichothecium* (4.75%), and *Valsa* (3.58%) were the main taxonomic groups in rotten fruit. The fungal compositions in rotten fruit were significantly different among different producing regions (Figure 1D). Since postharvest diseases in pear fruits are mostly caused by fungi, the main fungal components can be considered as pathogens that cause fruit rot. In Botou, the relative abundances of *Botryosphaeria*, *Colletotrichum*, and *Valsa* were significantly increased in rotten fruit. *Alternaria* and *Botryosphaeria* were dominant in the rotten fruit of Weixian. Fungi including *Rosellinia*, *Botryosphaeria*, *Fusarium*, and *Trichothecium* were found in high abundance in the rotten fruit of Shenzhou. In Xinji, *Alternaria* and *Trichoderma* were the main fungi, while *Talaromyces*, *Botryosphaeria*, *Colletotrichum*, *Fusarium*, and *Trichothecium* were dominant in Jinzhou.

### 2.2. Comparison of Microbial Diversity in Pear Fruit

**Figure 2:**
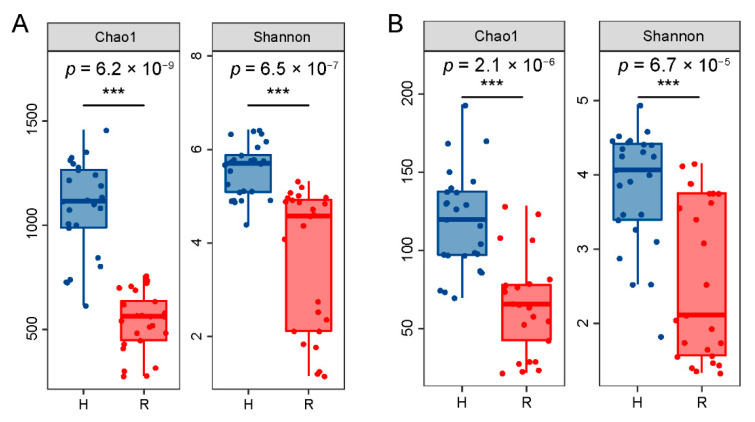
Alpha diversity (Chao1 and Shannon) analysis in healthy (H) and rotten (R) fruit of ‘Huangguan’ pear. (**A**), bacteria; (**B**), fungi. “***” represents significant differences (*p* < 0.05). H means healthy fruit (blue bar) while R means rotten fruit (red bar).

Chao1 and Shannon indices were used to characterize the microbial richness and diversity of healthy and rotten pear fruit. As shown in Figure 2, the richness and diversity of bacterial and fungal communities in rotten fruit were significantly lower (*p* < 0.05) than those in healthy fruit, indicating that dominant species in both fungal and bacterial communities appeared in rotten fruit.

**Figure 3:**
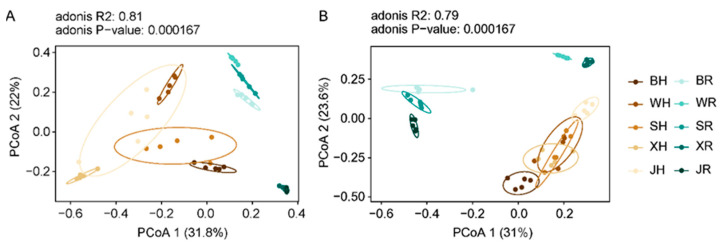
Principal coordinates analysis (PCoA) plots of Bray–Curtis dissimilarities in the bacterial (**A**) and fungal (**B**) communities of ‘Huangguan’ pear. Healthy fruit from Botou, Weixian, Shenzhou, Xinji, and Jinzhou was named BH, WH, SH, XH, and JH, while rotten fruit was named BR, WR, SR, XR, and JR, respectively.

The principal coordinates analysis (PCoA) showed that there were significant differences in the microbial diversity between healthy fruit and rotten fruit among all producing regions, indicating that differences exist in the relative abundance of certain microorganisms in rotten fruit, differentiating it from healthy fruit (Figure 3, Table 1). In addition, there were significant differences in the microbial diversity of fruit, both healthy and rotten, across all pear-producing regions, except that WH vs. SH and WH vs. XH had no significant differences in fungi, implying that fruit production regions have an impact on fungal and bacterial community diversity.

### 2.3. Correlation Analysis of Fungi and Bacteria

**Figure 4:**
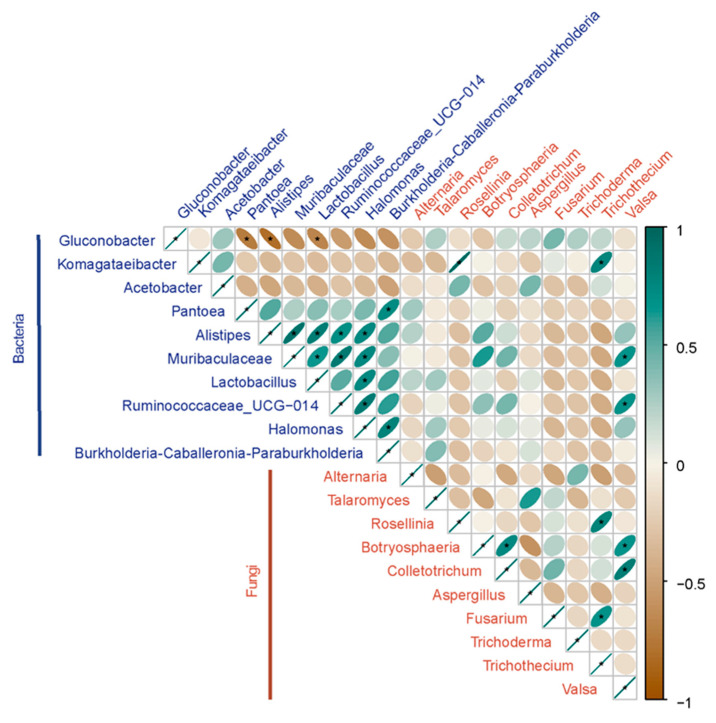
Correlation analysis of abundant bacteria and fungi in ‘Huangguan’ fruit.

To characterize the relationship between the dominant fungi and bacteria, the Pearson method was used for the analysis of microbial correlation. As shown in Figure 4, some bacteria and fungi are positively correlated (*p* < 0.05), indicating that they may have the potential to be functionally related. For instance, bacterial genus *Komagataeibacter* was highly correlated with fungal genus *Rosellinia* and *Trichothecium*. Similarly, *Muribaculaceae* and *Valsa*, and *Ruminococcaceae*_UCG-014 and *Valsa* were also positively correlated, respectively. In addition, some fungi are positively correlated with each other. *Botryosphaeria* and *Colletotrichum* were positively correlated. *Trichothecium* was positively correlated with *Fusarium* and *Rosellinia*. *Valsa* was positively correlated with *Colletotrichum* and *Botryosphaeria*. Similarly, some bacteria also have positive correlations with each other. On the other hand, the bacterial genus *Gluconobacter* was negatively correlated with *Pantoea*, *Alistipes*, and *Lactobacillus*, suggesting that there may be functional mutual exclusion between these bacteria, which needs further experimental confirmation.

### 2.4. Metabolic Pathways of Microorganisms

**Figure 5:**
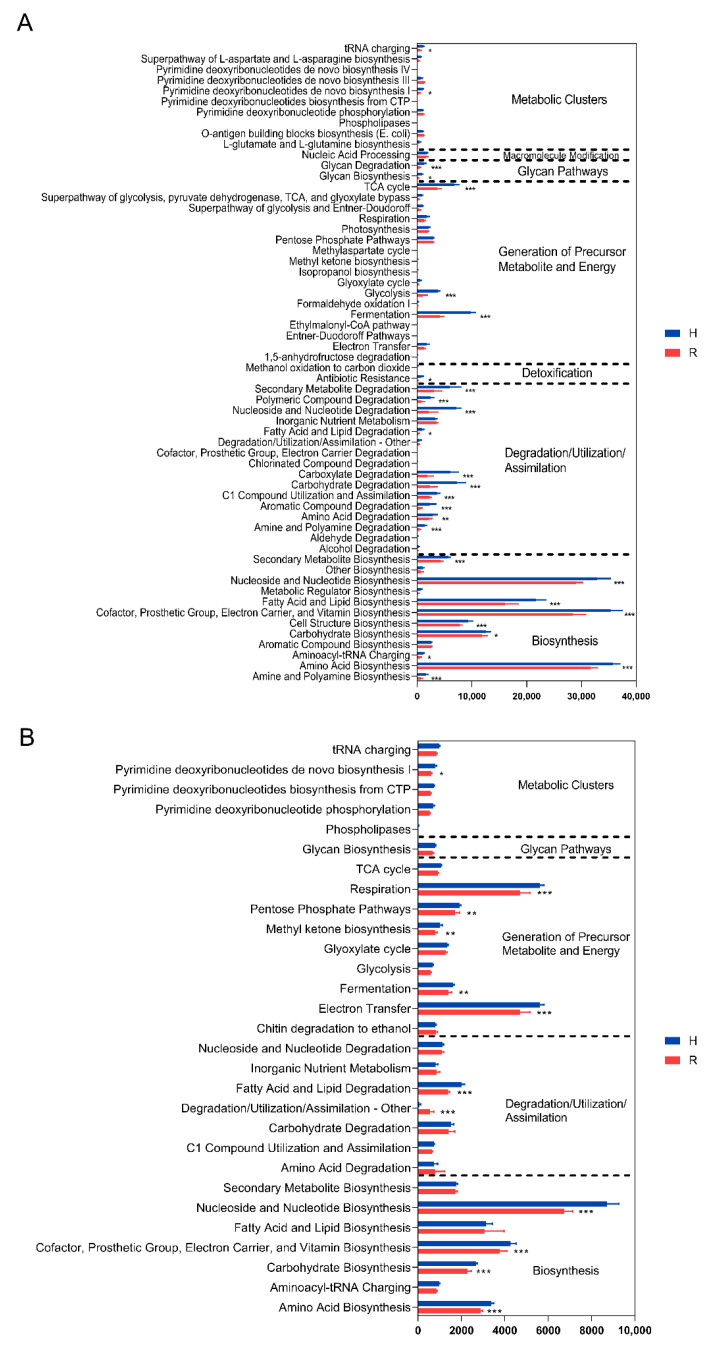
Bacterial (**A**) and fugal (**B**) metabolic pathways in healthy and rotten fruit of ‘Huangguan’ pear. “*”, “**”, and “***” represent the significant difference at *p* < 0.05, *p* < 0.01, and *p* < 0.001 levels, respectively.

To characterize the functional potential of the microbial community, we used PICRUSt2 to predict the metabolic pathways of the microorganisms in fruit. Figure 5 compares the summary statistics for the abundance of functional pathways in healthy and rotten fruit. From the figure, it can be seen that pathways including metabolic clusters, generation of precursor metabolite and energy, degradation/utilization/assimilation, and biosynthesis were significantly suppressed in the microbial community of rotten fruit for both bacteria and fungi. Glycan and detoxification pathways were decreased in the bacterial community of rotten fruit.

### 2.5. Mycotoxin Content and Its Relationship with Microbial Composition

Mycotoxins are secondary metabolites secreted by fungi, which seriously threaten human health. Here, we determined four mycotoxins, including PAT, AOH, AME, and TeA, in both healthy and rotten ‘Huangguan’ pear fruit. There were no mycotoxins detected in healthy fruit, while some mycotoxins were detected in rotten fruit.

**Figure 6:**
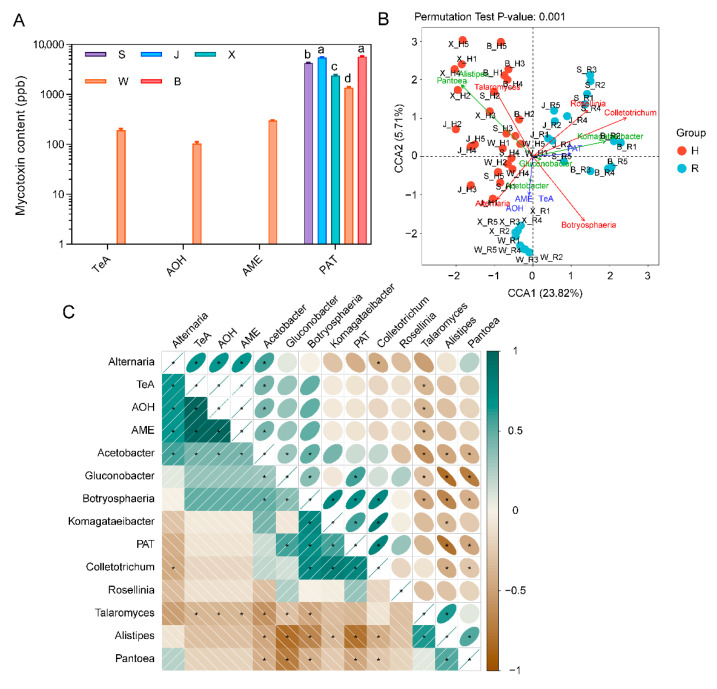
Relationship between mycotoxin production and microbial composition in ‘Huangguan’ pear fruit. (**A**) Mycotoxin content in rotten fruit; Values labeled with the different letters represent significant differences at *p* < 0.05; (**B**), Canonical correlation analysis; (**C**) Pearson correlation analysis. Bacteria are marked with green lines, while fungi are marked in red and mycotoxins in blue.

The results for the mycotoxin content in rotten fruit from various producing regions are presented in Figure 6A. The contents of four mycotoxins were determined, among which PAT was the highest. Indeed, Jinzhou and Botou had the highest PAT content, followed by Shenzhou, Xinji, and Weixian. The other three mycotoxins, TeA, AOH, and AME, were only detected in Weixian, but not in other producing regions, implying that different harvesting region had certain effects on the mycotoxins of rotten fruits during storage. The results from canonical correlation analysis (CCA) and correlation analysis showed that the abundance of *Alistipes* and *Pantoea* were negatively correlated with the content of PAT in rotten fruit, suggesting that bacterial genera *Alistipes* and *Pantoea* might play roles in the synthesis or degradation of mycotoxins in rotten fruit (Figure 6B,C). In addition, the contents of AOH, AME, and TeA were positively correlated with the abundance of *Alternaria* and *Acetobacter*. However, it is surprising that *Colletotrichum*, *Komagataeibacter*, *Botryosphaeria*, and *Gluconobacter* were positively correlated with PAT, and their functions need to be further studied.

## 3. Discussion

Fruit microorganisms have become an important object of fruit research, which can provide a new perspective for the prediction and control of postharvest fruit diseases [21]. In this study, microbial community diversity was investigated in healthy and rotten fruit of ‘Huangguan’ pear after 30 days of storage, and the relationship between mycotoxin content and microbial composition in rotten fruit was investigated as well.

The composition of fungi is closely related to fruit rot because most of the pathogens of postharvest fruit diseases are characterized as pathogenic fungi [25]. Fungi including *Botryosphaeria*, *Colletotrichum*, *Valsa*, *Alternaria*, *Rosellinia*, *Fusarium*, and *Trichothecium* were found to be abundant in rotten fruit, which were important pathogenic fungi causing postharvest diseases in fruits [26,27,28,29,30,31,32]. Interestingly, the fungal composition of rotten fruit varied significantly between different producing regions. Considering the different geographical locations of the five cities, the microbial diversity on the surface of pear fruit might be affected by the environmental factors, management patterns, and disease prevalence among them. In addition, *Trichoderma* was found to be abundant in Xinji in the rotten fruit, and studies have shown that *Trichoderma* was an antagonistic fungus and had inhibitory effects on pathogenic fungi in fruits and vegetables [33,34,35]. Therefore, *Trichoderma* may be involved in the inhibition of some fruit diseases, but may not be able to completely inhibit the occurrence of all diseases.

Bacterial composition analysis revealed that *Gluconobacter* abundance was significantly higher in rotten ‘Huangguan’ pear fruit than in healthy fruit; a similar result was also observed in our previous study [36]. Although bacteria in *Gluconobacter* genus have been reported to have antagonistic effects on fruit fungal diseases, most studies have shown that it can promote fruit rot and cause postharvest loss [37,38,39]. *Komagataeibacter* was found to be dominant in rotten fruit of Botou, which has been reported to produce cellulose and identified to be abundant in fruit fermentation [40,41,42]. The results of Pearson correlation between bacteria and fungi showed that *Komagataeibacter* was highly correlated with pathogenic fungi *Rosellinia* and *Trichothecium*, suggesting that there might be functional synergy between them. *Acetobacter* has a strong oxidative capacity, which can cause the decay of fruits and vegetables, as well as the deterioration of wine and fruit juices [43]. Large amounts of *Acetobacter* were determined in rotten fruit, indicating the important role it played during the storage of ‘Huangguan’ pear. In addition, *Pantoea* was identified to be more abundant in healthy fruit, indicating the positive role that it may play during the storage of pear fruit. Indeed, many studies have shown that *Pantoea* was very effective against pathogenic fungi and provided excellent control against plant diseases [44,45,46]. Therefore, *Pantoea* was likely to act as a guardian to protect the fruit from pathogen infection.

PAT was determined to be the highest mycotoxin in rotten ‘Huangguan’ pear fruit. Indeed, PAT exists in many kinds of rotten fruits with a high detection rate [47,48,49]. Due to the diffusion of PAT in fruit and its harmful effects on human health, consumers should avoid fruits with diseased spots [50]. Alternaria toxins including TeA, AOH, and AME were only detected in Weixian, indicating that Alternaria toxins were also present in rotten fruit, but not as widely as PAT. The relative abundance of *Alistipes* and *Pantoea* was negatively correlated with the contents of PAT, AOH, AME, and TeA in rotten fruits. *Alistipes* is mainly found in human gut microbiota, which has protective effects against some diseases, while it plays an opposite role in the occurrence of some other diseases [51]. *Pantoea* has shown great potential in antagonizing fungi and reducing mycotoxin production [52,53]. The content of *Pantoea* in healthy fruit was significantly higher, implying that *Pantoea* may play a role in inhibiting disease occurrence or toxin production in pear fruit. The function of *Pantoea* in pear fruit postharvest diseases is of great application significance, which is worthy of further study. A positive correlation was found between the content of AOH, AME, and TeA and the fungal abundance of *Alternaria*, indicating that the abundance of *Alternaria* could predict the content of Alternaria toxins.

## 4. Materials and Methods

### 4.1. Storage Conditions and Sample Preparation of Pear Fruit

The ‘Huangguan’ pear (*Pyrus bretschneideri* Rehd) fruit was harvested on 11 August 2020 from 15 orchards, and 3 orchards each of 5 cities including Botou, Weixian, Shenzhou, Xinji, and Jinzhou, Hebei Province, China. Fruit with similar size and maturity was selected and stored at ambient temperatures under 25 ± 1 °C, with a 90 ± 2% humidity.

After storage for 30 days, the fruit rot process occurred and their microorganisms were collected by homogenizing using a blender. Healthy fruits from Botou, Weixian, Shenzhou, Xinji, and Jinzhou were named BH, WH, SH, XH, and JH, respectively, while rotten fruits were named BR, WR, SR, XR, and JR, respectively.

Three healthy fruits were randomly selected, while rotten fruits with visible disease spots were selected. The fruits were then homogenized for 3 min using a blender, and 1 g of homogenate was collected in a sterile centrifuge tube, frozen with liquid nitrogen, and stored at −80 °C. Five replicates were set up in this study.

### 4.2. DNA Extraction and Amplicon Sequencing

The total DNA from each sample was extracted using a DNA kit (M5635-02, Omega, Norcross, GA, USA) and stored at −20 °C. The DNA of all samples was diluted to 20 ng/μL, and PCR amplification was carried out according to the following: 5 μL of 5 × Q5 reaction buffer, 5 μL of 5 × GC buffer, 2 μL of dNTP (2.5 mM), 1 μL of forward primer (10 μM), 1 μL of reverse primer (10 μM), 2 μL of DNA template, 8.75 μL of ddH_2_O, and 0.25 μL of Q5 DNA polymerase (M0491L, NEB, Ipswich, MA, USA).

The V5-V7 region of 16S rRNA gene was amplified with the primers 799F (5′-AAC MGG ATT AGA TAC CCK G-3′) and 1193R (5′-ACG TCA TCC CCA CCT TCC-3′), and the ITS1 region of the fungal community was amplified with the primers ITS1 (5′-CTT GGT CAT TTA GAG GAA GTA A-3′) and ITS2 (5′-GCT GCG TTC TTC ATC GAT GC-3′), combined with adapter and barcode sequences [36]. The thermal cycling condition was set as follows: 98 °C for 2 min; 30 cycles of 98 °C for 15 s, 55 °C for 30 s, and 72 °C for 30 s; and 72 °C for 5 min, 10 °C to hold, using the ABI 2720 PCR cycler machine (Thermo, Waltham, MA, USA). The sequencing of PCR products was carried out by Illumina MiSeq/NovaSeq platform at Personal Biotechnology, Shanghai, China.

### 4.3. Bioinformatic Pipeline for Analysis of Microbial Diversity

The raw sequences were processed using the DADA2 pipeline to generate the amplicon sequence variants (ASVs) [54]. The classify-sklearn function in QIIME2 software was used to annotate the obtained ASVs [55]. The Silva database (Release132, http://www.arb-silva.de (accessed on 7 December 2021)) was used for the annotation of the 16S rRNA gene [56], and the UNITE database (Release 8.0, https://unite.ut.ee/ (accessed on 9 December 2021)) was used for ITS sequences [57]. The rarefaction method was employed to normalize all samples at the same sequencing depth level, which was 95% of the sequences for the minimum sample [58,59].

Alpha-diversity metrics including Chao1 [60] and Shannon [61] were estimated using the diversity plugin with samples rarefied to the same number of sequences. Bray–Curtis dissimilarity was used for the beta diversity matrix to calculate the differences between samples [62]. Principal coordinate analysis (PCoA) was plotted using ImageGP [32]. Pearson correlations of fungi and bacteria and canonical correlation analysis (CCA) were carried out by using the genescloud tools (https://www.genescloud.cn/ (accessed on 12 December 2021)). Fungal and bacterial functions were predicted using the Phylogenetic Investigation of Communities by Reconstruction of Unobserved States (PICRUSt2) in the MetaCyc database (https://metacyc.org/ (accessed on 12 December 2021)).

### 4.4. Determination of Mycotoxin Production

Mycotoxins were extracted and determined according to Wang et al. [63] with minor modifications. Briefly, 5 g of homogenized fruit was dilute with Milli-Q water to 5 mL, followed by an addition of 20 mL of MeCN containing 100 mM citric acid. After shaking at 150 rpm for 30 min, 2 g of NaCl was added into the tube and centrifuged at 10,000 rpm for 5 min. Four milliliters aliquot of the upper MeCN layer was collected after passing through the SPE cartridge. The extract was evaporated at 50 °C under nitrogen stream, and resolved with 1 mL of MeCN/water (3/7, *v*/*v*) containing 5 mM NH4AC. The resulting solution was then forced through a 0.22 μM PTFE membrane filter (Pall, Westborough, MA, USA), and the content of mycotoxins was analyzed by UPLC/ESI-MS/MS.

A C18 column (ACQUITY CORTECS UPLC, Waters, Milford, MA, USA) was used for the separation of LC with the mobile phases containing 5 mM NH4AC (A) and MeCN (B) at a flow rate of 0.3 mL min^−1^. Positive and negative ionization modes were performed with the following parameters: capillary voltage at +2.5 kV/−1.5 kV; source temperature at 150 °C; desolvation temperature at 500 °C; cone gas flow at a rate of 150 L h^−1^; and desolvation gas flow at a rate of 1000 L h^−1^. The monitoring modes of multiple reactions were used for detection. The data were acquired and processed through MassLynx^TM^ software (v4.1 SCN937, Waters, Milford, MA, USA).

### 4.5. Statistical Analysis

Figures of alpha diversity, Pearson correlation, CCA, and PICRUSt2 analysis were created by using the genescloud tools (https://www.genescloud.cn/ (accessed on 12 December 2021)). The differences in microbial functions and mycotoxin content between healthy and rotten fruit were plotted by GraphPad Prism 8.0 software (GraphPad Inc., San Diego, CA, USA). Two-way analysis of variance (ANOVA) was used to show the significance of different groups. PerMANOVA was performed by ImageGP with the default parameters (http://www.ehbio.com/Cloud_Platform/front (accessed on 6 March 2022)).

## Figures and Tables

**Table 1:** Analysis of the composition of the bacterial and fungal communities in healthy fruit.

Pairs	Bacteria			Fungi		
	R^2^	*P*-Adjusted	Sig	R^2^	*P*-Adjusted	Sig
BH vs. WH	0.591978	0.011053	*	0.321574	0.011053	*
BH vs. SH	0.212455	0.0465	*	0.312754	0.011053	*
BH vs. XH	0.721622	0.011053	*	0.215719	0.015714	*
BH vs. JH	0.409482	0.011053	*	0.532116	0.011053	*
BH vs. BR	0.813081	0.011053	*	0.702342	0.011053	*
BH vs. WR	0.81491	0.011053	*	0.749095	0.011053	*
BH vs. XR	0.679522	0.011053	*	0.747734	0.011053	*
BH vs. JR	0.721766	0.011053	*	0.680768	0.011053	*
WH vs. SH	0.396408	0.011053	*	0.171005	0.055739	
WH vs. XH	0.71682	0.011053	*	0.162669	0.070833	
WH vs. JH	0.359256	0.011786	*	0.348047	0.011063	*
WH vs. BR	0.827208	0.011053	*	0.716855	0.011053	*
WH vs. WR	0.658041	0.011053	*	0.681124	0.011053	*
WH vs. SR	0.72838	0.011053	*	0.688894	0.011053	*
WH vs. XR	0.843609	0.011053	*	0.677885	0.011053	*
WH vs. JR	0.859374	0.01186	*	0.72708	0.011053	*
SH vs. XH	0.517982	0.011786	*	0.164089	0.027209	*
SH vs. JH	0.278789	0.011053	*	0.316119	0.011053	*
SH vs. BR	0.651772	0.011053	*	0.738717	0.011053	*
SH vs. WR	0.616787	0.011053	*	0.695862	0.011053	*
SH vs. SR	0.586405	0.011053	*	0.697443	0.011053	*
SH vs. XR	0.586903	0.011053	*	0.693377	0.011053	*
SH vs. JR	0.631411	0.011053	*	0.735392	0.011053	*
XH vs. JH	0.40517	0.016193	*	0.382327	0.011053	*
XH vs. BR	0.887939	0.011053	*	0.712806	0.011053	*
XH vs. WR	0.89198	0.011053	*	0.688394	0.011053	*
XH vs. SR	0.878855	0.011053	*	0.682479	0.011053	*
XH vs. XR	0.89183	0.011053	*	0.685562	0.011053	*
XH vs. JR	0.892796	0.011053	*	0.715241	0.011053	*
JH vs. BR	0.621556	0.011053	*	0.809491	0.011053	*
JH vs. WR	0.551114	0.011053	*	0.606817	0.011053	*
JH vs. SR	0.558436	0.011053	*	0.761766	0.011053	*
JH vs. XR	0.631405	0.011053	*	0.607294	0.011053	*
JH vs. JR	0.638654	0.011053	*	0.830115	0.011053	*
BR vs. WR	0.96884	0.011053	*	0.894688	0.011053	*
BR vs. SR	0.945308	0.011538	*	0.775024	0.011053	*
BR vs. XR	0.975101	0.011053	*	0.920598	0.011053	*
BR vs. JR	0.977461	0.011053	*	0.83395	0.011053	*
WR vs. SR	0.908071	0.011786	*	0.851352	0.011341	*
WR vs. XR	0.983926	0.011053	*	0.90034	0.011053	*
WR vs. JR	0.987103	0.011053	*	0.958482	0.011053	*
SR vs. XR	0.961185	0.011053	*	0.858435	0.011053	*
SR vs. JR	0.955762	0.011053	*	0.760137	0.011063	*
XR vs. JR	0.890881	0.011053	*	0.961663	0.011053	*

* means significant difference at the 5% level.

## Data Availability

Data of ITS and 16S amplicon sequencing of the samples were submitted to NCBI under the Bioproject Id PRJNA882998.
